# A Medical Plan of Campaign

**Published:** 1888-08-25

**Authors:** 


THE HOSPITAL

A WEEKLY INSTITUTIONAL JOURNAL OF

Science, Medicine, Nursing, and Philanthropy.

*For Hospitals, Asylums, Medical Practitioners, Students, Nurses, Families, Parishes, Congregations, and the Charitable Public.*

VOL. IV. No. 100.] [AUGUST 25, 1888.

### A MEDICAL PLAN OF CAMPAIGN.

THE Swedish Commission which has been appointed to investigate the nature, cure, and prevention of tuberculosis is setting about its work with energy and good sense. The Commissioners are not satisfied with making the inquiry a mere doctor's question. Recognizing the fact that it is the public much more than the medical profession whose interests are at stake, they are about to publish a series of popular papers on the subject with the view of enlisting the active sympathy and help of all intelligent persons, whether lay or medical, in their important and difficult labours. This is entirely as it should be. The idea that members of the general public have nothing whatever to do with the progress of medicine and surgery is ludicrous. It would be just as reasonable to assert that they have nothing to do with the harvest because they are not farmers, or with the fashions because they are not tailors. If medicine and surgery do not concern the public, whom do they concern? It is the public who take the potions of the doctors, who submit to all kinds of dangerous surgical operations, who consent to be killed or cured *secundum artem.* As a matter of fact, the subject of tuberculosis is to the doctor a question for scientific speculation, or for use as a stepping-stone to fame, or to the successful practice of his bread-winning art. But to large multitudes of the general public it is a question of life and death.

The Swedish Commission have shown how superior is their intelligence and how much broader is their comprehension of their public duties than that displayed by many who fain would be leaders of medical thought and progress in this country. We cannot but reiterate the expression of our contempt and just anger against those who desire to make the medical profession a close corporation, a trades unionism, a professional monopoly, an arcana of mysteries into which none but the initiated have a right even to cast a curious and inquiring eye. Medicine, like every other art and science, must come out into the full light of open day and of public criticism. No art or science has less to fear from such criticism than modern medicine and surgery. They who bring the clear light of day to bear upon medical science are its best friends, whilst of all its enemies there are none so dangerous as those members of its own household who build thick walls around it and guard its portals against all intrusion from a critical outside world. "No admission except on business" may be a proper motto for those who have doubtful or dangerous occupations to conceal. But the free spirit of modern medicine flings wide the gate in order that all the world may enter and behold her honest and zealous labours for the general health and well-being.

The medical profession throughout the whole world is bestirring itself with an almost unprecedented activity to discover if possible the cause or causes of tubercular phthisis, the range and limits of its operations, the methods of neutralising its potency, and of combatting its effects when once established. The campaign, for such it is, must be conducted by the scientific and trained professors of medicine; but men of every class have the fullest right and claim to help in every possible way—by inquiry, by suggestion, by criticism, by monetary aid. The practically International Congress, whose sittings have taken place in Paris, has rendered services of the highest value in this world-wide cause. Some of the labours of the Congress tend to show in the most convincing way the harmlessness of the tubercle bacillus under certain conditions. Here, for example, is a case in point. M. Strauss exhibited to the members of the Congress several fowls which had for varying periods been fed on tubercular expectorations. Those expectorations are generally virulent, and in these particular cases their virulence had been proved by inoculations of guinea pigs. The inoculated animals had become the subjects of phthisical disease. Two of the fowls fed on the expectorations for nearly twelve months had taken no harm whatever. They were killed in the presence of members of the Congress and their organs carefully examined. The lungs and other viscera were found to be in perfect health, and the general condition of the fowls was excellent. One of them had eaten as much as a hundred pounds weight of phthisical sputa in twelve months.

Now what is the special significance of these experiments? They show almost to a demonstration that tubercle bacilli may be present in the blood of vertebrate animals for months together, without harm —unless we assume, which is in the highest degree improbable, that the expectoration taken as food had all passed through the alimentary canal unchanged, or that the gastric juice had proved poisonous to all the bacilli and their spores. Such assumptions, however, hardly need be entertained for a moment. It cannot be doubted that the phthisical expectoration which was eaten almost daily for several months was digested without the entire destruction of its virus, taken up by the blood and lymph, circulated freely in these fluids through every part of the body, passed through the glands of the lungs and elsewhere, and otherwise performed the functions of nutrient matter. All this took place for ten or twelve months without the slightest injury of any kind to certain of the fowls experimented upon. Yet fowls can be infected with phthisis without difficulty, though there is no reason to suppose they are so easily infected as man.

An experiment of this kind tends to show that there are limits to the poisonous efficacy of the tubercle bacillus. These limits may be more circumscribed than has been supposed. To have proved that the tubercle bacillus may be digested by a warm-blooded animal, and that in the process of digestion it is rendered innocuous, or that it is only dangerous to tissues in a state of peculiar susceptibility is a great step in advance. It does not show us anything in the way of a cure; but it adds to our knowledge of the nature and limitations of the efficient agent in the disease. It seems to show, moreover, that a peculiar susceptibility of the tissues involved in an attack of phthisis is an essential condition of successful infection. This, it may be remarked, is quite in accord with universal experience, and with the beliefs which are generally prevalent on the subject.

A curious antagonism, which has long been believed to exist between malarial poison and the poison of the tubercle bacillus, has received marked confirmation from members of the Congress. It seems certain that the inhabitants of marshy and malarious countries are much less subject to consumption than those who live in districts free from malaria. There appears, however, no reason to suppose that persons already infected with phthisis can be cured by removal to marshy districts. A more evidently practical point on which some light has been thrown is the degree of vitality displayed by the tubercle germ under varying circumstances. In carefully sterilised running water taken from the Seine the bacillus and its spores were alive at the end of fifty days; in water similarly prepared, but kept at rest, they were still alive at the end of a hundred and twenty days.

All these experiments are of the nature of investigations into the cause and progress of phthisis rather than of its cure. But directly curative attempts have repeatedly been made for centuries, and with how little result! It is wise therefore now to do what, perhaps, ought to have been done long ago, and to begin at the beginning. There seems to be more than a possibility that the character, life history, and potency of the tubercle bacillus will soon become matters of general knowledge, at least among members of the medical profession. These circumstances once well-known, it will be strange, indeed, if some fortunate experiments do not light upon a method either of curing the disease when established, or of so increasing the resisting power of the susceptible tissues as to make them proof against its ravages. In any case, the first step to be taken in conquering a formidable enemy is to find out all about his strength and his weakness, and to take measures in accordance with the undoubted facts.

